# Production of Adult Human Synovial Fluid-Derived Mesenchymal Stem Cells in Stirred-Suspension Culture

**DOI:** 10.1155/2018/8431053

**Published:** 2018-03-27

**Authors:** Kristen D. Jorgenson, David A. Hart, Roman Krawetz, Arindom Sen

**Affiliations:** ^1^Pharmaceutical Production Research Facility (PPRF), Schulich School of Engineering, University of Calgary, 2500 University Drive NW, Calgary, AB, Canada T2N 1N4; ^2^McCaig Institute for Bone and Joint Health, Cumming School of Medicine, University of Calgary, 3280 Hospital Drive NW, Calgary, AB, Canada T2N 4Z6

## Abstract

The chondrogenic potential of synovial fluid-derived mesenchymal stem cells (SF-MSCs) supports their use in cartilage regeneration strategies. However, their paucity in synovial fluid necessitates their proliferation in culture to generate clinically relevant quantities. Here it was determined that 125 mL stirred suspension bioreactors utilizing Cytodex-3 microcarrier beads represent a viable platform for the proliferation of these cells. During the inoculation phase, a bead loading of 2 g/L, an inoculation ratio of 4.5 cells/bead, and continuous agitation at 40 rpm in a medium with 5% serum resulted in high cell attachment efficiencies and a subsequent overall cell fold expansion of 5.7 over 8 days. During the subsequent growth phase, periodic addition of new microcarriers and fresh medium increased culture longevity, resulting in a 21.3 cell fold increase over 18 days in the same vessel without compromising the defining characteristics of the cells. Compared to static tissue culture flasks, a bioreactor-based bioprocess requires fewer handling steps, is more readily scalable, and for the same cell production level, has a lower operating cost as it uses approximately half the medium. Therefore, stirred suspension bioreactors incorporating microcarrier technology represent a viable and more efficient platform than tissue culture flasks for the generation of SF-MSCs in culture.

## 1. Introduction

Articular cartilage is a connective tissue that covers the ends of bones, providing load absorption and dissipation, and a near friction-free surface that enables bones to articulate within a joint. The avascular nature of cartilage and the low density of dispersed chondrocytes (cartilage-producing cells) greatly hinder the endogenous regenerative capacity of this tissue [1]. As such, even slight damage to cartilage can initiate the development of osteoarthritis (OA) in which cartilage degeneration is significant and results in joint swelling, chronic pain, and reduced mobility [2].

OA has traditionally been treated by administering pharmaceuticals to alleviate symptoms such as pain [3]. However, pharmaceuticals can lose their efficacy over time, result in significant undesirable side effects, and have not yet been shown to be able to maintain or regenerate cartilage [4–7]. Thus, many patients eventually have no choice but to undergo surgery [8]. In extreme cases, total joint replacement (TJR), in which the damaged joint is replaced by a prosthetic joint, is necessary. Although TJR can improve patient quality of life, patients do not completely regain normal function, and issues related to infection and joint loosening over time suggest that alternative treatments are required [7].

Newer treatment options that have been tested include transplanting plugs of cartilage isolated from non-weight-bearing areas to the defect site (mosaicplasty) [1, 5]. However, this approach can result in donor site morbidity, and methods to fix the new cartilage to the defect site, such as sutures and pins, may actually initiate further damage [9]. A second approach has been to expand, in culture, populations of chondrocytes isolated from a cartilage biopsy for subsequent implantation into a defect site, sometimes in conjunction with biomaterials (autologous chondrocyte transplantation) [5, 6]. This approach can also result in donor site morbidity, and the use of biomaterials is not desirable [10]. Moreover, chondrocytes have limited expansion capacity in culture and tend to dedifferentiate and lose their ability to make cartilage [11]. A third method has been to drill through the subchondral bone, resulting in the release of marrow elements and the subsequent formation of a blood clot in the defect site, which, through natural healing mechanisms, is typically replaced over time by a fibrous type of cartilage [1, 6]. This fibrocartilage does not have the mechanical properties or durability of native articular cartilage [6, 12, 13].

Mesenchymal stem cells (MSCs) have recently generated considerable interest for their potential to repair cartilage. These cells can be isolated from several different sources, including bone marrow, adipose tissue, and synovial fluid. Adult human MSC populations are defined by their surface marker profile (CD34^−^, CD45^−^, CD73^+^, CD90^+^, and CD105^+^), their capacity to attach to cell culture-grade plastic, their ability to generate colonies, and their trilineage potential to become fat, bone, or cartilage cells [14]. Despite having these characteristics in common, MSCs are influenced by the tissue microenvironment in which they reside, and thus, MSC populations from different tissues exhibit specific traits which serve to distinguish them from one another [15, 16].

MSCs isolated from within articulating joints have shown a superior capacity to contribute to cartilage repair. For example, significant efforts have been made to examine the possibility of using synovial membrane-derived mesenchymal stem cells for cartilage tissue engineering [9, 10, 17–23]. Synovial fluid-derived MSCs (SF-MSCs) are believed to originate from the synovial membrane but exist in the lubricating fluid contained within the joint cavity [24–26]. However, presumably due to local environmental influences, SF-MSCs have shown a greater capacity to generate cartilage than other evaluated MSC types, including those from synovial membrane, bone marrow, and adipose tissue [16, 27, 28]. Interestingly, during development, articular cartilage and synovial joint components are reported to be derived from progenitor interzone cells [29], and thus, adult MSCs in synovial membrane and synovial fluid may retain some of this cellular bias. This cell type has also been reported to possess robust growth potential [30]. SF-MSCs are easily harvested in a minimally invasive manner through arthrocentesis, thereby avoiding donor site morbidity [30]. Given that SF-MSCs can be derived from a very accessible source, they clearly represent a potentially valuable cell type for certain tissue engineering applications, including the repair of articular cartilage [30, 31].

Despite their accessibility, the low concentration of SF-MSCs in synovial fluid means that they cannot be isolated in sufficient numbers for the direct development of clinical repair and regeneration strategies. Moreover, if approved for therapeutic use, widespread clinical implementation of SF-MSC-based therapies will require large quantities of quality-assured cells. For these reasons, it is necessary to develop rapid and reproducible methods for the scaled-up expansion of these cells. The vast majority of stem cell research is carried out on cell populations which have been expanded in static tissue culture flasks. However, the use of tissue culture flasks for scale-up is not desirable. Due to the small culture volumes that can be accommodated in a single flask, many flasks are needed to generate clinically relevant numbers of cells making this approach for cell expansion inefficient and manually intensive. In contrast, bioreactors are scalable vessels that have been shown to be capable of supporting the expansion of a number of stem cell types [32–35] and thus represent a viable alternative to static culture flasks. A single suspension culture bioreactor can be designed to hold the same culture volume as hundreds of flasks, easily operated by a single trained individual, and computer controlled to continuously maintain an optimum and homogenous culture environment for the growth of cells. An important consideration when scaling up adherent cells in bioreactors is that they require a surface onto which they can attach to survive, grow, and proliferate, without concomitant differentiation. Microcarriers are small beads which can be introduced into a stirred bioreactor and maintained in suspension through agitation, thereby providing a surface for cell attachment and enabling the expansion of adherent cells in this dynamic environment [36, 37]. The use of microcarriers is not without its challenges, however, and studies are required to develop protocols which are customized to support both the attachment of a particular cell type to a specific microcarrier type and the proliferation of that particular cell type on the microcarrier.

In this report, studies were performed to determine the feasibility of culturing adult human SF-MSC populations in suspension bioreactors. It is shown here that microcarrier technology can be used to support the expansion of these cells without compromising their defining properties.

## 2. Materials and Methods

### 2.1. Static Culture

MSCs derived from the synovial fluid of two cadaveric male donors (donor 1 was 71 years old and donor 2 was 34 years old) showing no signs of OA were acquired within four hours of death via the Southern Alberta Tissue Transplant Program with approved ethics and consent protocols. The MSCs were isolated by standard methods [38]. Cryopreserved SF-MSCs at passage 2 were thawed and inoculated into 75 cm^2^ Nunc tissue culture flasks (T-75) at a density of 5000 cells/cm^2^ with Dulbecco's Modified Eagle's Medium (DMEM) (Lonza Cat number 12-707F). DMEM was supplemented with Mesenchymal Stem Cell Growth Medium (MSCGM) SingleQuot Kit (Lonza Cat number PT-4105) which contained fetal bovine serum (FBS). This complete medium was referred to as 10% FBS DMEM. The complete medium was stored at 4°C for a maximum of 2 weeks. Cultures were incubated at 37°C and 5% CO_2_, and a 50% medium change was performed after 3 days. Cultures were passaged every 5 days by harvesting the cells with 0.05% trypsin EDTA (Invitrogen Cat number 25300-120) and reinoculating them into new T-75 flasks at 5000 cells/cm^2^. Cell density and viability were assessed with a haemocytometer using the trypan blue exclusion method.

### 2.2. Suspension Culture

#### 2.2.1. Spinner Flask Preparation

Suspension culture expansion was carried out in 125 mL (maximum working volume) spinner flasks (NDS Technologies, NJ, USA) equipped with a suspended magnetic impeller. The inner surface of each spinner flask and its impeller were siliconized with Sigmacote (Sigma Cat number SL-2) to minimize cell and microcarrier attachment. All spinner flasks were fully assembled and autoclaved prior to use. During operation, each spinner flask was placed in a humidified incubator (37°C, 5% CO_2_) on top of a Thermolyne magnetic stir plate which was used to control the stirring rate of the impeller.

#### 2.2.2. Microcarrier Preparation

Cytodex 3 microcarriers (Sigma Cat number C3275, Lot number 030M1182V), which are dextran beads coated with denatured porcine-skin collagen on their surface, were chosen based on preliminary small-scale experiments carried out in our laboratory showing that human synovial fluid-derived MSCs can attach to these beads (data not shown). The microcarriers were prepared for use according to the manufacturer's specifications. Briefly, a known quantity of dry beads was hydrated in Ca^2+^- and Mg^2+^-free phosphate-buffered saline (PBS) overnight in an Erlenmeyer flask and then rinsed with fresh Ca^2+^- and Mg^2+^-free PBS prior to being sterilized in an autoclave. Microcarriers were prepared for immediate use only.

#### 2.2.3. Spinner Flask Inoculation

Sterilized microcarriers were rinsed with DMEM and introduced into spinner flasks with 60 mL of filtered cell culture medium. The spinner flasks were then incubated at 37°C and 5% CO_2_ for approximately 18 hours before being inoculated with cells. The cells used for inoculation were harvested from static tissue culture flasks and used to generate a cell suspension that was added to the spinner flasks for a total working volume of 80 mL. After 24 hours, all spinner flasks were topped up with an additional 40 mL of cell culture medium to a final working volume of 120 mL. Great care was taken to ensure consistency in inoculation between replicate flasks.

#### 2.2.4. Spinner Flask Sampling

At designated time points during each experiment, four representative 1000 *μ*L samples were taken from each spinner flask with a calibrated 1000 *μ*L pipette. Prior to each sample being taken, the flask contents were well mixed, and the sample was obtained from the centre of the culture volume. This aided in maintaining consistency between samples and in ensuring that the obtained samples were representative of the flask contents. Each sample was placed in a single, sterile 15 mL conical tube and left undisturbed in order to allow the microcarriers to settle. Once settled, the supernatant was discarded, and the microcarriers were rinsed twice with 1.0 mL of PBS (each rinse involved the addition of PBS to the microcarriers, followed by removal once the microcarriers had settled). Next, 1.0 mL of 0.1% (*w*/*v*) crystal violet in 0.1 M citric acid was added to each conical tube, and the microcarriers were incubated for 1 hour at 37°C. After 1 hour, the microcarrier suspension was agitated 10 to 15 times with a 1000 *μ*L pipette. A 20 *μ*L aliquot of the stained cell suspension was removed, and the released nuclei were counted with a haemocytometer as a measure of the culture cell density.

To visualize the cells on the microcarriers, a 500 *μ*L sample from a spinner flask was placed in a well of a 6-well plate with 3.0 mL of PBS and 15 *μ*L of 0.5% (*w*/*v*) crystal violet in methanol, and the sample was examined under a Zeiss Axiovert 200 microscope.

#### 2.2.5. Harvesting Cells from Microcarriers

To harvest cells from Cytodex 3 microcarriers, a 10 mL sample from a spinner flask was placed in a 15 mL conical tube, and the microcarriers were rinsed twice with 4.0 mL of PBS. A volume of 1.0 mL of 0.05% trypsin-EDTA was then added to the conical tube, and the contents were gently agitated 5 times with a 1000 *μ*L pipette. After allowing the microcarriers to settle (2 minutes), the supernatant containing the detached cells was collected with a pipette and passed through a BD Falcon 100 *μ*m cell strainer (VWR Cat number CA21008-950) into a 50 mL conical tube. The addition of 1.0 mL of trypsin-EDTA to the microcarriers followed by gentle agitation and filtration of the supernatant into the same conical tube was repeated twice more. The filter was rinsed three times with 1.0 mL of culture medium, and the accumulated filtrate was centrifuged at 600 ×g for 5 minutes to pellet and isolate the cells.

### 2.3. Cell-Surface Marker Analysis

The prevalence of MSC-specific surface antigens was determined by flow cytometry. Briefly, cells were rinsed twice with PBS and incubated in blocking solution (3% FBS in PBS) on ice in the dark for 30 minutes. Cells were centrifuged and resuspended in blocking solution at a density of 5 × 10^5^ cells/100 *μ*L and distributed into 100 *μ*L aliquots in 15 mL conical tubes. Each 100 *μ*L aliquot was stained with 5.0 *μ*L of antibodies against human CD34, CD45, CD73 (BD Biosciences Cat number 550822, 555483, and 550257, resp.), CD90, and CD105 (Serotec Cat number MCA90F and MCA1157F, resp.). Following 30 minutes of incubation on ice in the dark, cells were rinsed three times with PBS, resuspended in blocking solution, and transferred to BD Falcon round-bottom tubes (VWR Cat number CA60819-138). Relative fluorescence was measured using a FACSCalibur flow cytometer (BD Biosciences), and data were analyzed with CellQuest software.

### 2.4. Differentiation

Cells were induced towards osteogenic, adipogenic, and chondrogenic fates with commercially available differentiation induction and maintenance media kits from Lonza. Differentiation protocols were carried out according to the manufacturer's instructions and are briefly described here.

#### 2.4.1. Osteogenic Differentiation

Osteogenesis was induced in 6-well plates using an osteogenic differentiation kit (Lonza Cat number PT-3002) which included osteogenic induction medium (OIM). Cells were plated at a density of 3.0 × 10^4^ cells/well (3.1 × 10^3^ cells/cm^2^) with 3.0 mL of 10% FBS DMEM. After 24 hours, the 10% FBS DMEM was discarded and replaced with OIM for the duration of the differentiation period. Cells were maintained in culture for 28 days and complete medium changes were performed every 3 days. Alizarin Red was used to stain for calcium deposition in osteogenic cultures as previously described [37, 39].

#### 2.4.2. Adipogenic Differentiation

Adipogenesis was induced in 6-well plates using an adipogenic differentiation kit (Lonza Cat number PT-3004) which included adipogenic induction medium (AIM) and adipogenic maintenance medium (AMM). Cells were plated at a density of 2.0 × 10^5^ cells/well (2.1 × 10^4^ cells/cm^2^) with 3.0 mL of 10% FBS DMEM. Complete medium changes were performed every 2-3 days with 10% FBS DMEM until the cells were 100% confluent, generally after 5 days. Once confluent, the 10% FBS DMEM was replaced with 3.0 mL of AIM for 3 days before being replaced with 2.0 mL of AMM for 2 days. This 5-day cycle with AIM and AMM was repeated two more times. At the end of the third cycle, cells were maintained in AMM for the remainder of the 28-day differentiation period with complete medium changes every 3 days.

Oil Red O (ORO) solution was used to stain for lipid droplet formation in adipogenic cultures. The stock solution of ORO was prepared by adding 0.175 g of ORO (Sigma Cat number O0625) to 50 mL of 100% isopropanol. The working solution of ORO was then prepared by adding 60% (*v*/*v*) ORO stock solution to 40% (*v*/*v*) double-distilled water. The staining procedure was carried out as previously described [37, 39].

#### 2.4.3. Chondrogenic Differentiation

Chondrogenesis was induced using the pellet culture method with a chondrogenic differentiation kit (Lonza Cat number PT-3003) and transforming growth factor *β*3 (TGF-*β*3) (Lonza Cat number PT-4124). The chondrogenic induction medium (CIM) from the kit was referred to as incomplete CIM (iCIM) until the TGF-*β*3 was added at which point it was complete CIM (cCIM). Each pellet was generated using 2.5 × 10^5^ cells in a 15 mL conical tube. Cells were centrifuged and rinsed with iCIM (300 × g for 5 minutes). The iCIM was discarded and the pellet was resuspended in cCIM. The pellets were maintained in culture using cCIM for 28 days with complete medium changes every 3 days.

To quantify the extent of chondrogenesis, the glycosaminoglycan (GAG) content was measured. The medium was discarded and the pellets were transferred to 0.7 mL Eppendorf tubes and digested in a 65°C water bath for 4 hours with 50 *μ*L papain solution (12.5 mg of papain (Sigma Cat number P4762) and 16.32 mg of N-acetyl-2003L-cysteine (Sigma Cat number A9165) in 50 mL of 50 mM phosphate buffer). The pellets were vortexed and then centrifuged at 1000 rpm for 1 minute every 30 minutes. To ensure complete digestion, vigorous agitation with a 1000 *μ*L pipette to completely break up the pellet was necessary prior to a final centrifugation at 3000 rpm for 5 minutes. The supernatant was isolated and evaluated for GAG content.

A standard curve was generated by serial dilution of 10 mg of chondroitin sulphate (Sigma Cat number C9819) dissolved in 1.0 mL of 50 mM phosphate buffer. The quantity of GAGs present in a given sample or standard solution was based on a color reaction with a dimethylmethylene blue (DMB) solution (8 mg of 1,9-dimethylmethylene blue (Sigma Cat number 341088) in 2.5 mL of ethanol mixed with 500 mL double-distilled water containing 1.0 g sodium formate and 1.0 mL formic acid). 10 *μ*L of either standard solution or sample was placed in the wells of a 96-well plate with 200 *μ*L of DMB solution and the plate was incubated at 37°C and 5% CO_2_ for 30 minutes before being analyzed. All standards and samples were measured in triplicate with a plate reader at 510 nm.

### 2.5. Statistical Analysis

Data were statistically analyzed using one-way ANOVA. A *p* value less than 5% (*p* < 0.05) was considered significant.

## 3. Results and Discussion

Suspension bioreactors represent a scalable platform to generate large numbers of cells in culture in an efficient and reproducible manner [35, 37, 40]. However, when dealing with adherent cells and microcarrier technology, there are two distinct operating phases which need to be considered. The first is the inoculation phase during which conditions are created to encourage the inoculated cells to attach to the microcarriers. The second is the growth phase where cells attached to microcarriers are encouraged to proliferate. Conditions which are ideal for cell attachment may not necessarily be optimal to support cell proliferation [41]. Thus, to maximize cell yield, there is a need to better understand and optimize each phase of this bioprocess. Here, the results from studies concerning both phases are discussed. Cells from donor 1 were used to develop a suspension bioreactor protocol, and then cells from both donors were used to evaluate the final protocol to ensure that the methodology was not specific to cells from a single donor.

### 3.1. The Inoculation Phase

The inoculation phase of adherent cell types, particularly in microcarrier suspension cultures, can have a significant influence on the ultimate cell population yields [41–43]. Inoculation phase culture parameters such as agitation regimen, serum levels in the medium, microcarrier loading (g/L), and cell to bead ratio have all been shown to affect cell attachment efficiency.

Forestell et al. [41] indicated that after attachment, cells remained rounded on the microcarrier surface prior to spreading and taking on a flatter profile. These rounded cells were more susceptible to detachment than cells which had spread when exposed to the shear created by continuously stirring the medium. Yuan and colleagues [37] reported that a period of 8 hours was sufficient for attachment of bone marrow-derived mesenchymal stem cells to macroporous CultiSpher-S microcarriers. However, Hewitt et al. [44] reported that following inoculation, 24 hours was required for this same cell type to attach and then spread on microporous Cytodex 3 microcarriers. Thus, we initially defined the inoculation phase as being the first 24 hours following the addition of human SF-MSCs to the bioreactors.

As a starting point, inoculation protocols recommended by the microcarrier manufacturer in combination with media and cell densities used in traditional static culture flasks were used. The baseline parameters were as follows: (i) continuous stirring at 40 rpm which was the minimum agitation required to maintain the microcarriers in suspension throughout the volume of medium, (ii) a 10% serum level at inoculation which mimics static culture, (iii) 4.5 cells/bead which mimics static culture at 5000 cells/cm^2^, and (iv) Cytodex 3 microcarrier loading of 1 g/L. The inoculation phase was carried out in a reduced culture volume of 80 mL. After 24 hours, the cultures were topped up to a final working volume of 120 mL in preparation for the growth phase. Culture parameters used following the first 24 hours included continuous stirring at 40 rpm and 10% serum in the culture medium.

We microscopically evaluated cell attachment and spreading behaviours in response to the manipulation of several different culture parameters (agitation regimen, initial serum content, microcarrier loading, and cell to bead ratio) during the 24-hour inoculation phase. However, accurate quantification of attachment efficiency during this period was challenging due to the low cell numbers in the bioreactor. Thus, we instead assessed the impact of manipulating a particular inoculation phase parameter by measuring cell numbers that resulted in culture after 10 days (24-hour inoculation phase plus a subsequent 9-day growth phase). During the growth period, culture conditions were maintained consistent across all vessels to isolate any effects associated with the inoculation phase. This technique was deemed appropriate as higher cell attachment efficiencies during the inoculation phase have been directly correlated to higher subsequent cell yields [41–43]. Thus, measuring cell yield provided us with a means to evaluate cell attachment efficiency in response to a particular inoculation parameter.

#### 3.1.1. Effect of Agitation Regimen

Literature reports of agitation rates employed during the inoculation of mesenchymal stem cells into suspension culture range from 0 rpm (i.e., no stirring) for the first 24 hours [43, 44] to continuous stirring at 30 rpm for the first 18 hours [36]. Others have reported using an intermittent agitation regimen consisting of alternating periods of stirring and rest [45–47]. Forestell and colleagues [41] found that cell attachment occurs less frequently at higher agitation rates. As such, they used the minimum agitation speed required to suspend the microcarriers. In those studies where intermittent agitation was reported, cultures were agitated for anywhere from 5 seconds to 30 minutes followed by 10 to 30 minutes of rest.

**Figure 1:**
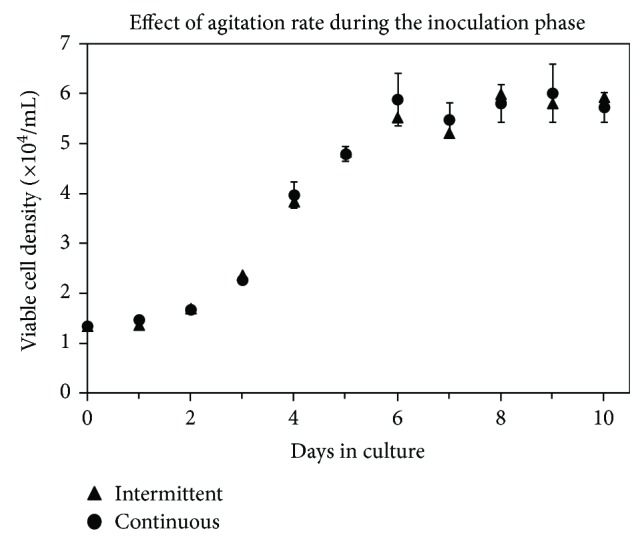
Effect of agitation regimen on the attachment of SF-MSCs to Cytodex 3 microcarriers during the inoculation phase. During the first 24 hours, cultures were either agitated at 40 rpm for 3 minutes every 30 minutes (Intermittent) or continuously at 40 rpm (Continuous). Data were collected in duplicate; error bars represent the range of data collected.

In the current study, cultures were stirred at 40 rpm either continuously or intermittently with cycles of 3 minutes of agitation followed by 27 minutes of rest. It has previously been shown that this regimen facilitated the attachment of bone marrow-derived MSCs to microcarriers [37]. The resulting growth curves are shown in Figure 1. The exponential growth rate was 0.0126 h^−1^ for both continuous and intermittent cultures. The maximum cell densities reached were 6.1 × 10^4^ and 6.0 × 10^4^ cells/mL for the continuous and intermittent agitation regimen, respectively.

From this study, it appeared that there was no significant difference in SF-MSC attachment efficiency to Cytodex 3 microcarriers when using continuous versus intermittent agitation. This is not an unusual result as successful attachment of human placental MSCs on Cytodex 3 microcarriers [44] and bone marrow-derived MSCs on Cytodex 1 microcarriers [36] has been reported with continuous agitation of 30 to 50 rpm during the first 18 to 24 hours. We have noted that MSCs from synovial fluid tend to attach more readily to cell culture plastic than other MSC types in the presence of serum, and this tendency may have translated to the interactions between SF-MSCs and microcarriers in suspension culture. Based on this result, all future experiments in this study incorporated continuous stirring at 40 rpm during the first 24 hours.

#### 3.1.2. Effect of Serum Level

**Figure 2:**
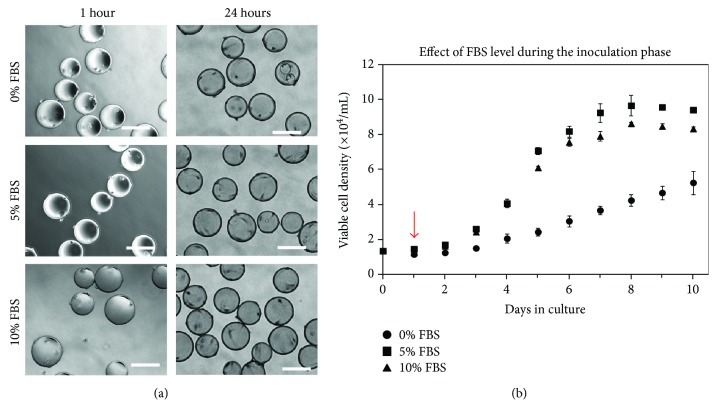
(a) Photomicrographs showing the effect of initial serum content on the attachment of SF-MSCs to Cytodex 3 microcarriers. Cell-microcarrier contact occurred within 1 hour of inoculation for all three serum levels evaluated. 24 hours after inoculation, many cells in 0% FBS were still rounded on the surface compared with 5% and 10% FBS where cells had spread. Photomicrographs were taken at 10x magnification. Scale bars represent 200 *μ*m. (b) Effect of initial serum content on the attachment of SF-MSCs to Cytodex 3 microcarriers and subsequent cell population expansion. Cells were cultured in 0%, 5%, or 10% serum for the first 24 hours. After 24 hours, as indicated by the arrow, the serum levels were adjusted to 10% *v*/*v* in all cases. Data were collected in duplicate; error bars represent the range of data collected.

The effect of culture medium serum level (0%, 5%, or 10% with respect to volume) during the inoculation phase was studied as it has been shown to impact attachment efficiency of bone marrow-derived mesenchymal stem cells (BM-MSCs) to microcarriers [36]. It should be noted that all microcarriers used in this study had been exposed to culture medium containing the respective serum content for a period of 18 h prior to being placed in the spinner flask (i.e., the 5% and 10% scenarios had been precoated with serum). Moreover, after the 24-hour inoculation phase, all spinner flasks were topped up to a final working volume of 120 mL medium so that the final concentration of FBS was 10% in all cases during the growth phase.  Figure 2(a) illustrates similar cell attachment regardless of serum level during the first hour after inoculation, with cells in all cases appearing rounded on the surface of the beads. However, after 24 hours, most of the cells in the 5% and 10% serum conditions had spread, whereas many of the cells in 0% serum were still rounded on the surface.

The results presented in Figure 2(b) show that the 0% serum condition during inoculation did not subsequently support cell growth as effectively as the 5% and 10% conditions. Not only did 0% serum at inoculation result in a prolonged lag phase, but after 10 days, the exponential growth rate of 7.75 × 10^−3^ h^−1^ was significantly lower when compared with 0.0159 h^−1^ and 0.0160 h^−1^ for 5% and 10% serum, respectively. The maximum cell density obtained over the course of 10 days in the 0% serum inoculated condition was also significantly lower than that for both the 5% and 10% cases. The maximum cell density in the 5% serum was higher than the maximum cell density achieved with 10% serum. The maximum density in 5% serum was 9.66 × 10^4^ cells/mL compared to 8.62 × 10^4^ cells/mL in 10% serum.

Schop et al. [36] and Forestell et al. [41] both reported higher attachment efficiencies at 0% to 5% (*v*/*v*) serum levels for the attachment of mammalian cells on Cytodex 1 microcarriers compared to 10% serum levels. They speculated that serum lowers surface hydrophobicity, which could negatively impact cell attachment. Conversely, it has been reported that precoating Cytodex 2 microcarriers (cross-linked dextran matrix) with fetal bovine serum decreased the fraction of unoccupied beads 7 hours after inoculation, thereby improving attachment efficiency [48]. FBS is known to contain many different proteins, such as fibronectin and albumin. It has been suggested that when beads are exposed to FBS, albumin readily adsorbs first, and this could interfere with cell attachment, but that over time, this albumin is replaced with fibronectin, which promotes cell attachment [48]. The precoating of microcarriers with serum has also been shown to enhance the attachment of other cell types including human bone marrow-derived MSCs on CultiSpher-S beads [49]. This current result clearly illustrates that simply changing one condition during the inoculation phase can have a significant impact on subsequent cell yields. Based on the results obtained, subsequent experiments used culture medium containing 5% serum to precoat the microcarriers for 18 hours and also for the duration of the inoculation phase.

#### 3.1.3. Effect of Microcarrier Loading and Cell to Bead Ratio

Cell to bead ratio is another important factor that affects ultimate bioreactor cell yields as it directly impacts the frequency of cell-bead interactions, a necessary prelude to cell attachment. Under ideal circumstances, the initial cell to bead ratio should be unity, if it could be ensured that each bead would only allow for the attachment of a single cell. However, during the inoculation phase, the number of cells per microcarrier has been shown to follow a Poisson distribution [41, 42, 45]. As a result, some microcarriers will have more than one cell attached to their surface, while a portion of the microcarriers may not be occupied by any cells at all. Thus, to ensure that a majority of beads are occupied at the end of the inoculation phase, it is necessary to have a cell to bead ratio greater than unity. According to the Poisson distribution, the proportion of vacant microcarriers is theorized to be less than 2% when the cell to bead ratio equals or exceeds a value of 4.

To investigate the effect of cell to bead ratio and microcarrier loading on the attachment of SF-MSCs on Cytodex 3 microcarriers, a two-level factorial design experiment was carried out. Microcarrier loadings of either 1 g/L or 2 g/L Cytodex 3 were used, which correspond to available surface areas of 2.7 cm^2^/mL and 5.4 cm^2^/mL, respectively. The cell to bead ratio selected as the baseline value was 4.5 cells/bead, which is equivalent to 5000 cells/cm^2^ typically used to inoculate static tissue culture flasks. A lower cell to bead ratio of 2.25 cells/bead was also evaluated. Whereas this could result in a greater proportion of unoccupied beads at the end of the inoculation phase, a low inoculation density could result in a greater overall cell fold increase. Cell to bead ratios higher than 4.5 were not evaluated since this would require a larger number of cells for inoculation and could potentially result in a lower cell fold increase as a significant proportion of the cells could fail to attach and subsequently perish, thereby wasting inoculum.

**Figure 3:**
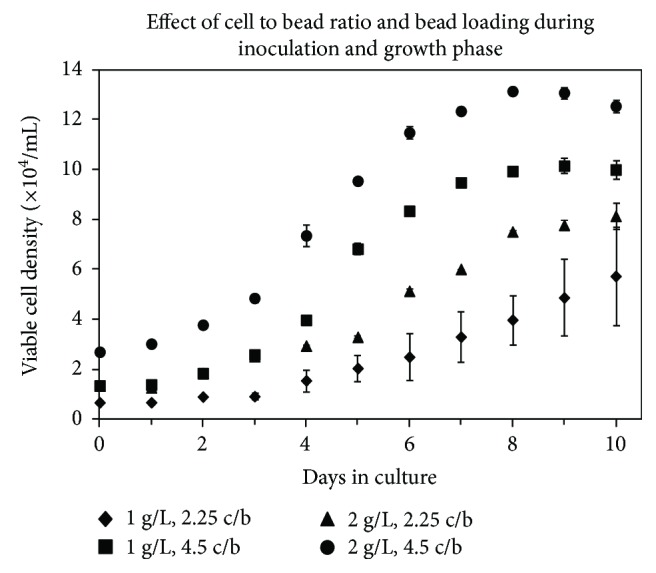
Effect of microcarrier loading and cell to bead ratio on the attachment of SF-MSCs to Cytodex 3 microcarriers. Spinner flasks were inoculated with either 0.12 g (1 g/L) or 0.24 g (2 g/L) of Cytodex 3 at a cell density of either 2.25 or 4.5 cells/bead (c/b). Data were collected in duplicate; error bars represent the range of data collected.

As shown in Figure 3, it was found that at both 1 g/L and 2 g/L microcarrier loading, a seeding density of 2.25 cells/bead does not reach final cell densities comparable to those achieved using a seeding density of 4.5 cells/bead. Additionally, at a seeding density of 2.25 cells/bead and a microcarrier loading of 1 g/L, a 3-day lag phase was observed. These trends are similar to those reported in the study by Hu et al. [42] where it was reported that a critical number of cells per microcarrier is required for a normal pattern of growth. The growth rates during the exponential phase for 2.25 cells/bead at 1 g/L and 2 g/L were similar at 0.0103 h^−1^ and 0.0102 h^−1^, respectively. The exponential growth rates using 4.5 cells/bead were 0.0159 h^−1^ and 0.0118 h^−1^ for 1 g/L and 2 g/L, respectively. The decrease in growth rate at higher microcarrier loadings has previously been observed for other cell types and in those cases has been attributed to an increase in bead-to-bead collisions resulting in cell damage and death [50]. Here relatively high cell viabilities were maintained in culture, and significant quantities of cell debris were not observed during the growth phase, suggesting that the increased frequency of collisions at high bead loadings may reduce growth and proliferation without necessarily killing cells. A positive effect of such collisions, however, is the transfer of cells from one bead to another. As expected, a majority of the microcarriers had at least one cell attached when microscopically analyzed after 24 hours. However, during the growth phase, the number of empty microcarriers observed decreased suggesting that cells had transferred from a bead with cells to an empty bead, either during a bead-bead collision or through a process of detachment into the surrounding medium and then reattachment.

Based on the shortened lag phase and increased growth rates obtained when using 4.5 cells/bead, this ratio was selected for all future experiments. Despite the higher growth rate at 1 g/L, an initial microcarrier loading of 2 g/L was selected for future studies as it (i) supports cell proliferation, (ii) provides a surface area to volume ratio that is twice that of 1 g/L of beads and is similar to that used in static culture vessels (generally between 4.2 and 6.1 cm^2^/mL), and (iii) supports twice the theoretical maximum volumetric cell density (cells/mL) of a 1 g/L bead loading, which is an important consideration as it means that twice as many cells have the potential to be produced within a given vessel.

### 3.2. Growth Phase

Once favourable parameters had been established for the inoculation phase, variables that were hypothesized to affect cell population expansion during the growth phase were evaluated. Specifically, the effects of agitation rate and periodic medium replenishment were investigated in an effort to identify their impact on cell growth kinetics. In these experiments, the inoculation parameters selected (continuous agitation at 40 rpm, 5% serum culture medium, 4.5 cells/bead, and a microcarrier loading of 2 g/L) were applied to all cultures for the first 24 hours. At the end of the inoculation phase, various agitation rates and feeding patterns were tested throughout the growth phase.

#### 3.2.1. Effect of Agitation Rate

Agitation in microcarrier-based suspension culture has several effects: (i) it creates a more homogeneous culture environment, (ii) it introduces shear stress into the culture environment, (iii) it provides collision energy between microcarriers, and (iv) it increases mass transfer [41]. While a more homogeneous environment and increased nutrient transport may be beneficial for the culture of cells, high shear stresses can serve to damage cells attached to surfaces, promote the detachment of cells from the surface of a microcarrier, and result in strong collisions between microcarriers leading to cell death [50]. It has been shown that a shear stress as low as 6.25 dyn/cm^2^ may be sufficient to remove cells from surfaces, with 10–30 dyn/cm^2^ resulting in damage to cells [51, 52]. Since shear stress and collision energy are related to bioreactor agitation rate, stirring speed was manipulated to evaluate the impact of shear on SF-MSC proliferation. Preliminary experiments showed that a low agitation rate of 40 rpm in the spinner flasks was sufficient to maintain Cytodex 3 microcarriers in suspension and also provide a homogenous, well mixed environment (data not shown).

It has been reported from aggregate studies with baby hamster kidney (BHK) cells that the maximum shear stress experienced by cells at the surface of an aggregate ranges from 0.8 dyn/cm^2^ to 5.6 dyn/cm^2^ for agitation rates of 25 rpm to 100 rpm [52]. Previous studies in our laboratory involving neural stem cell aggregates in the same spinner flask bioreactors being used in the current study found that the shear stress experienced by a single cell at the surface of an aggregate ranged from 4.9 dyn/cm^2^ to 9.86 dyn/cm^2^ for agitation rates from 40 rpm to 100 rpm ([53]; Gilbertson et al., 2006). Since these shear stress values are all less than those which have been reported to cause damage to a cell immobilized to an aggregate surface or static surface, agitation rates of 40 rpm, 60 rpm, and 80 rpm were evaluated in this study.

**Figure 4:**
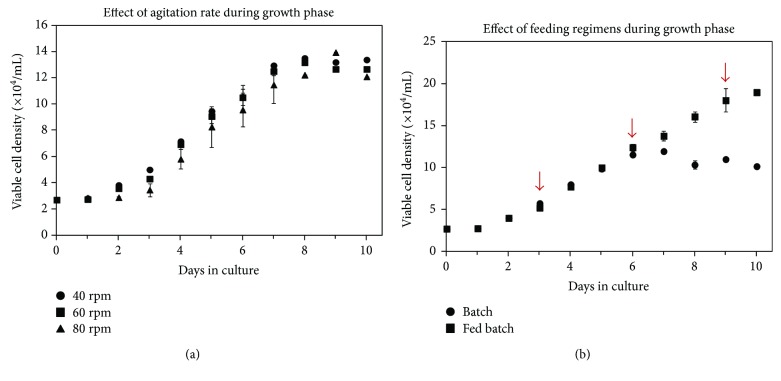
(a) Effect of agitation rate on the growth of SF-MSCs on Cytodex 3. Six spinner flasks were stirred continuously at 40 rpm for the first 24 hours. After 24 hours, the agitation rate in two spinner flasks was increased to 60 rpm and in another two was increased to 80 rpm. Data were collected in duplicate; error bars represent the range of data collected. (b) Effect of feeding on cell growth. In the cyclic fed batch condition, a 50% medium change was performed on days 3, 6, and 9, indicated by the arrows. No medium replenishments were made in the batch condition. Data were collected in duplicate; error bars represent the range of data collected.

Although there was no evidence of significant differences in proliferation between any of the agitation rates tested, a slightly longer lag phase was observed at 80 rpm (Figure 4(a)). It is likely that the sudden increase from 40 rpm during the inoculation period to 80 rpm at the start of the growth phase led to some cells detaching from the beads. At 40 rpm, 60 rpm, and 80 rpm, the exponential phase growth rates were 0.0128 h^−1^, 0.0123 h^−1^, and 0.0126 h^−1^, respectively, and the maximum cell densities obtained were very similar between the three agitation rates. However, the maximum cell density at 80 rpm occurred later than at the lower agitation rates, presumably because of the extended lag phase. The similarity in cell expansion at the different agitation rates indicates that shear was not a significant factor affecting proliferation rate in the range tested. In addition, there was no noticeable difference in the level of cell debris in the culture medium at the end of the experiment, suggesting that cells were not being destroyed at any of the tested agitation rates. Given that significant differences were not found, an agitation of 40 rpm was used in all subsequent studies as this could facilitate the transition from the inoculation phase to the growth phase.

#### 3.2.2. Effect of Feeding Regimen

The rapid expansion of cells in culture can result in the depletion of key nutrients such as glucose and glutamine from the medium, while simultaneously increasing the levels of waste metabolites including lactate and ammonium ions. The absence of nutrients and the presence of wastes may negatively impact ultimate cell yields. There are numerous examples in the literature showing that regular medium changes, in which a portion of the spent culture medium is removed and replaced with an equal volume of fresh medium, can extend the productive life of a culture and increase cell yields, including those of bone marrow-derived MSC cultures [46].

To determine if medium components were limiting SF-MSC growth in suspension culture, the effect of medium replenishment was evaluated by culturing cells in either batch culture, where no medium replenishments were made, or in cyclic fed-batch cultures, where a 50% medium replenishment was performed on days 3, 6, and 9. Growth curves generated over a 10-day period for batch and cyclic fed-batch conditions are shown in Figure 4(b). Due to initial similar growth patterns, the exponential growth rates for the batch and cyclic fed-batch cultures (from day 2 to day 6) were both 0.0136 h^−1^. However, feeding extended the life of the culture, thereby allowing a greater overall cell yield. The maximum cell density achieved in the cyclic fed-batch culture was 1.89 × 10^5^ cells/mL (day 10), which was significantly higher than that achieved in the batch culture, 1.19 × 10^5^ cells/mL (day 7). This translated to a viable cell fold increase of 7.0 over 10 days under cyclic fed-batch conditions, compared to only 4.4 over the same time period under batch conditions. It is important to note that these results also showed that the microcarrier surface area was not limiting in batch culture and that the lack of cell growth beyond day 6 was likely due instead to nutrient depletion or waste accumulation in the medium.

Nutrient and metabolic waste product concentrations were monitored throughout the 10-day growth period for batch and cyclic fed-batch cultures. SF-MSC glucose and glutamine consumption rates during the growth phase in batch cultures were calculated as 3.88 × 10^−10^ mmol/cell·h and 2.41 × 10^−10^ mmol/cell·h, respectively. These rates are comparable to the values obtained in a previous study that found the average glucose consumption rate of human bone marrow MSCs to be 3.83 ± 0.69 × 10^−10^ mmol/cell·h [54]. Additionally, lactic acid and ammonium production rates during the growth phase in the batch cultures were 1.95 × 10^−9^ mmol/cell·h and 1.74 × 10^−10^ mmol/cell·h, respectively. The yield of lactic acid over glucose (*Y*_Lac/Glc_) during the growth phase in the batch cultures was 2.94 mol/mol. A value higher than the theoretical maximum, 2 mol/mol, indicates that lactate is being produced from other sources (i.e., glutamine) [49]. *Y*_Lac/Glc_ values ranging from 1.4 to 6.5 mol/mol have been reported previously for mesenchymal stem cells [36]. The yield of ammonia from glucose was 0.689 mol/mol. As little as 2.0 mM of ammonia has been reported to inhibit proliferation of human MSCs, whereas lactic acid concentrations upwards of 24 mM may be required before inhibitory effects become apparent [54]. Based on the concentration profiles, the concentration of ammonia in batch culture approached 2.0 mM after 6 days in culture, while the concentration of ammonia in all cyclic fed-batch cultures did not exceed 1.5 mM (data not shown). Since the nutrient profiles showed that glucose and glutamine were not depleted, it is possible that inhibitory levels of ammonia may have played a role in the reduced proliferation observed in batch culture, as the maximum cell density was also achieved on day 6. Another possibility is that the addition of fresh medium replenished other medium components, such as amino acids [55], which were depleted but not measured in this study, thereby contributing to higher cell densities in cyclic fed-batch cultures.

Based on the results of this study, a 50% medium replenishment approximately every 3 days was incorporated into the standard protocol for maintenance of human SF-MSCs in suspension culture.

### 3.3. Serial Subculture on Microcarriers

Whereas the feeding study clearly indicated that the lifespan of a culture could be extended by replacing spent medium with fresh medium, the yield of cells would ultimately be limited by the finite surface area provided by the microcarriers for monolayer growth. To increase cell yields beyond that point, two strategies were investigated: (i) serial passaging of microcarrier cultures and (ii) bead-to-bead transfer. In the serial passaging method, cells were harvested from microcarriers using trypsin and then reinoculated into new culture vessels with fresh microcarriers and culture medium. In this approach, the total number of cells produced would increase over time as either the cells would be inoculated into an increasing number of vessels or into larger vessels capable of supporting greater culture volumes. In the bead-to-bead transfer method, fresh microcarriers were added to an existing culture vessel and conditions manipulated to encourage cell migration from confluent beads to new, unoccupied beads within the same vessel. Successful bead-to-bead transfer has previously been reported for porcine MSCs [45], goat MSCs [46], and human MSCs [36, 47] grown on a variety of microcarriers. Assuming the success of both methods, one major advantage of bead-to-bead transfer over serial passaging of microcarrier cultures is that the cultures could be maintained in a single vessel for an extended period of time with minimal cell handling. Not only would this reduce the number of times the cells are exposed to the proteinase trypsin, but it also would lower the probability of contamination. Additionally, in the event a vessel becomes too small to support a target cell number, the entire culture volume could be transferred to a larger bioreactor along with fresh beads without the need to first trypsinize the cells from the existing beads.

#### 3.3.1. Serial Passaging

**Figure 5:**
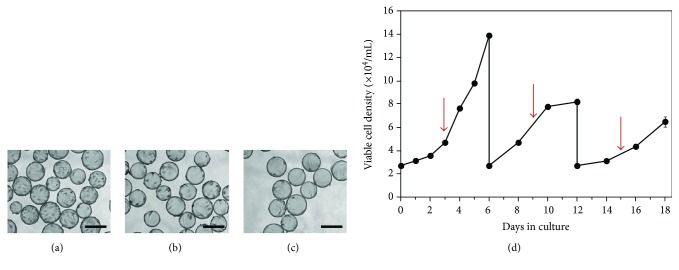
Serial passaging of SF-MSCs on Cytodex 3 microcarriers using the improved protocol. Cells were serially passaged 3 times. Each passage lasted 6 days. (a–c) Photomicrographs showing microcarriers from the last day of culture for three consecutive passages. There are fewer cells and a larger proportion of unoccupied beads with each subsequent passage. Photomicrographs were taken at 10x magnification. Scale bars represent 200 *μ*m. (d) Serial passaging of SF-MSCs on Cytodex 3. Lower maximum cell densities were achieved with each subsequent passage. A 50% medium replenishment was performed on days 3, 9, and 15, as indicated by the arrows. Data were collected in duplicate; error bars represent the range of data collected.

SF-MSCs expanded to passage 5 in static tissue culture flasks were inoculated into microcarrier culture and exposed to the inoculation and growth protocols described earlier. After six days, the cells were harvested from the microcarriers by trypsinization and inoculated into new vessels containing fresh microcarriers for three consecutive passages (6 days each) under the same conditions. Figures 5(a)–5(c) show photomicrographs taken on the sixth day of culture for three consecutive passages. With each subsequent passage, fewer total cells and a larger proportion of unoccupied beads were observed. Cell densities were plotted over the course of the three passages in Figure 5(d). Although the data shows that the harvested cells were able to reattach to microcarriers, they were unable to proliferate to the same degree as cells that had been harvested from static tissue culture flasks.

Hu et al. [56] reported serial propagation of human foreskin fibroblasts on Sephadex microcarriers (dextran beads). Although the cells used in the current study were able to reattach to new microcarriers and continue to grow, the extent of proliferation was found to decrease slightly with each subsequent inoculation. Similar results were described by Forestell and colleagues [57] for the serial subculture of human fetal lung fibroblasts on Cytodex 1 microcarriers. They found significant decreases in cell growth with each transfer from vessel to vessel. Forestell et al. [57] were able to overcome diminishing proliferation by decreasing the serum content and developing a custom medium supplement. It would be prudent in the future to examine the use of defined growth media to examine how serum levels impact SF-MSC behaviour during serial subculture. Analysis of the spent culture medium over the three serial passages indicated that glucose and glutamine were not depleted at any time, and waste metabolite concentration was also maintained at low levels (data not shown). As such, the diminished growth was likely not nutrient related.

#### 3.3.2. Bead-to-Bead Transfer

The effectiveness of bead-to-bead transfer of SF-MSCs on Cytodex 3 microcarriers during suspension culture was evaluated. Although the protocol developed in the previous sections used a microcarrier loading of 2 g/L, cultures were initiated with only 1 g/L for this particular study as microcarriers would be added periodically to the cultures, and thus, the benefit of a high surface area to volume ratio would eventually be achieved regardless of the initial bead loading. Moreover, a microcarrier loading of 1 g/L was shown to be effective earlier, and a smaller initial loading also meant that fewer cells were required as inoculum. Spinner flasks were initially inoculated with 4.5 cells/bead and a microcarrier loading of 1 g/L in 80 mL of 5% FBS DMEM and stirred continuously at 40 rpm for the first 24 hours. After 24 hours, spinner flasks were topped up with an additional 40 mL of medium and the serum levels were adjusted to 10% FBS DMEM. Every 6 days, 1 g/L of fresh microcarriers was added to the cultures, and culture volumes were reduced to 80 mL and stirred at 40 rpm intermittently for 3 hours (3 minutes on, 27 minutes off). Intermittent agitation was chosen because the work by Wang and Ouyang [58] involving Vero cells (i.e., African green monkey kidney cells) on Cytodex 3 showed that this operational mode enabled microcarriers to be in close proximity long enough for cells to effectively migrate from one bead to another. Whereas bead-to-bead transfer is also possible through collisions while in suspension, this latter approach can be traumatic and lead to cell damage and cell death. After 3 hours of intermittent agitation, cultures were stirred continuously at 40 rpm. The spinner flasks were topped up with an additional 40 mL of culture medium 24 hours after the microcarrier addition. 50% medium replenishments were performed on days 6, 9, 12, 14, and 16.

**Figure 6:**
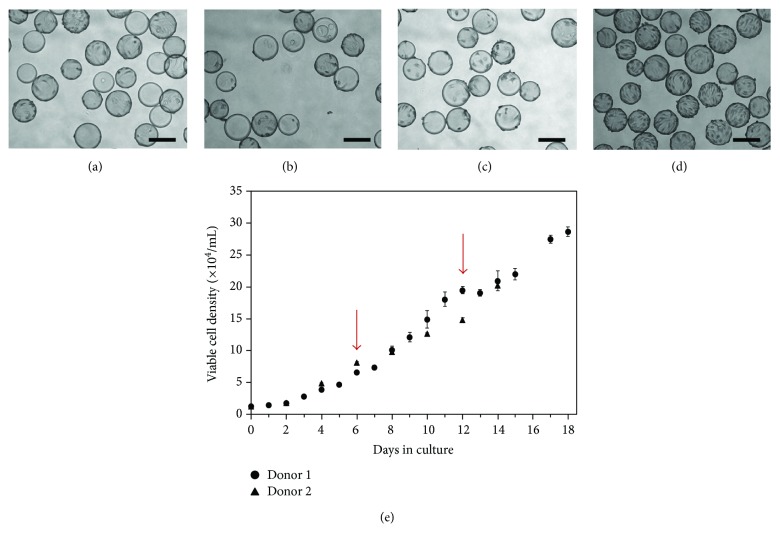
Bead-to-bead transfer of SF-MSCs on Cytodex 3 microcarriers. 1 g/L of fresh microcarriers was added to the culture on days 6 and 12 (shown with arrows). After addition, the cultured were stirred intermittently at 40 rpm for 3 hours (3 minutes on, 27 minutes off). After 3 hours, the culture was continuously stirred at 40 rpm. Shown are photomicrographs (a) immediately following addition of fresh microcarriers (day 6). Roughly half of the beads are empty and half are occupied and nearing confluence; (b) 3 hours after microcarrier addition on day 6. Cells had started to transfer to new microcarriers, but still appeared rounded on their surface; (c) 24 hours after microcarrier addition. More noticeable cell transfer had occurred and very few beads remained unoccupied; (d) 6 days following inoculation (day 12, prior to second addition of fresh microcarriers). Most beads were near confluence. Photomicrographs were taken at 10x magnification. Scale bars represent 200 *μ*m. (e) Growth curve of bead-to-bead transfer of SF-MSCs on Cytodex 3 microcarriers. Shown are data for cells derived from donors 1 and 2 to show that the utility of the protocol developed was not unique for only a single set of cells. The increases in viable cell densities show that the bead-to-bead transfer method was successful. Data were collected in duplicate; error bars represent the range of data collected.

Intermittent agitation was stopped after 3 hours because photomicrographs showed that many of the new beads had acquired cells after this time (Figures 6(a)–6(d)). Twenty-four hours after the addition of fresh microcarriers, the proportion of remaining empty beads decreased further compared to 3 hours postmicrocarrier addition. Similar to the observations noted earlier during the inoculation phase studies, this again suggests that cells were also able to transfer between microcarriers during periods of continuous agitation. The transfer of human BM-MSCs on Cytodex 3 beads during agitation was also observed by Hewitt et al. [44]. Cell adhesion is weakened during cell division and it is possible that it was during mitosis that cells were able to detach from their original microcarrier and reattach to a new bead [59].

Growth curves for cells from two donors are shown in Figure 6(e). The steady increase in viable cell densities shows that the bead-to-bead transfer method together with feeding is a viable method to enable prolonged cell growth in a single vessel. Additionally, a stationary phase was not reached suggesting that cell yields could have increased further had the experiment been allowed to continue. After 18 days in culture, a maximum cell density of 2.87 × 10^5^ cells/mL was achieved for donor 1, which translates to a 21.3-fold increase in the number of cells. Additionally, the volume of medium required per new cell generated was only 1.28 × 10^−5^ mL which was nearly half of what was required for each new cell generated in static culture (2.40 × 10^−5^ mL) over the course of 18 days. These values represent the total medium requirement, including medium changes. Therefore, not only was this method more cost-effective, but it also overcame the need to regularly expose cells to enzymes and significantly reduced labour requirements as frequent passaging was not necessary. Based on the studies presented thus far, it was evident that microcarrier technology could be used to effectively scale-up human SF-MSCs in suspension bioreactors. However, a series of characterization studies were needed to ensure that this mode of cell expansion had not adversely affected the defining qualities of these cells.

### 3.4. Cell Characterization after Expansion in Suspension Culture

SF-MSCs expanded for 18 days in suspension culture using the bead-to-bead transfer method were harvested and then characterized in terms of surface marker profile and differentiation potential to determine if they had retained their defining characteristics. Uninduced cultures, as well as cells grown under static conditions, were used as controls.

#### 3.4.1. Cell-Surface Marker Profile

**Figure 7:**
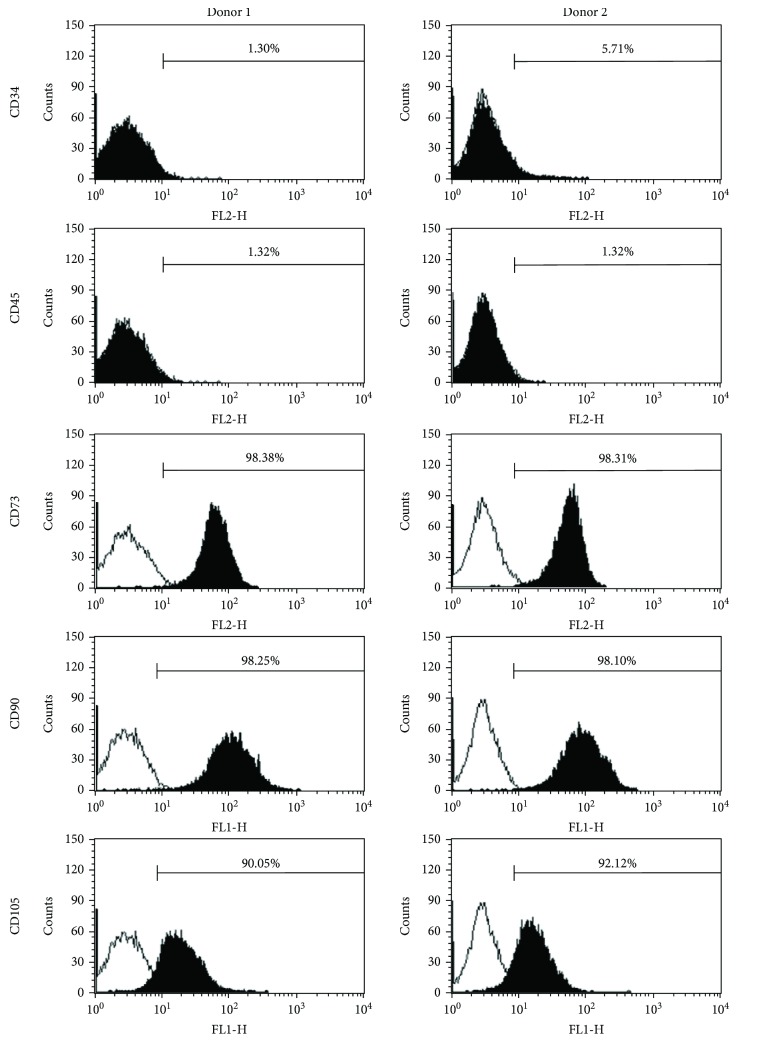
CellQuest histogram plots for surface markers associated with SF-MSCs. The plots show specific antibody staining (solid) versus isotype control staining (empty). Prior to analysis, cells were cultured for 18 days in spinner flasks using the bead-to-bead transfer method. A background noise level of 2% or less was accepted.

The surface marker panels analyzed were CD34, CD45, CD73, CD90, and CD105. This selection was based on the criteria outlined by Dominici et al. [14] which state that an MSC population must express CD73, CD90, and CD105 and must lack expression of CD34 and CD45. The surface marker profiles of donors 1 and 2 are presented in Figure 7 and indicate that both donors had high expression levels for positive makers and low expression levels for negative markers after being cultured using our optimized protocols.

#### 3.4.2. Differentiation Potential

Multipotency assays were performed to verify that cell populations expanded on microcarriers under stirred conditions using the newly developed protocols retained their capacity to undergo trilineage differentiation. Several other researchers have previously shown that bone marrow-derived MSCs grown on CultiSpher-S and Cytodex 1 and 3 microcarriers retain their osteogenic and adipogenic differentiation potential [36, 37, 47, 49].

**Figure 8:**
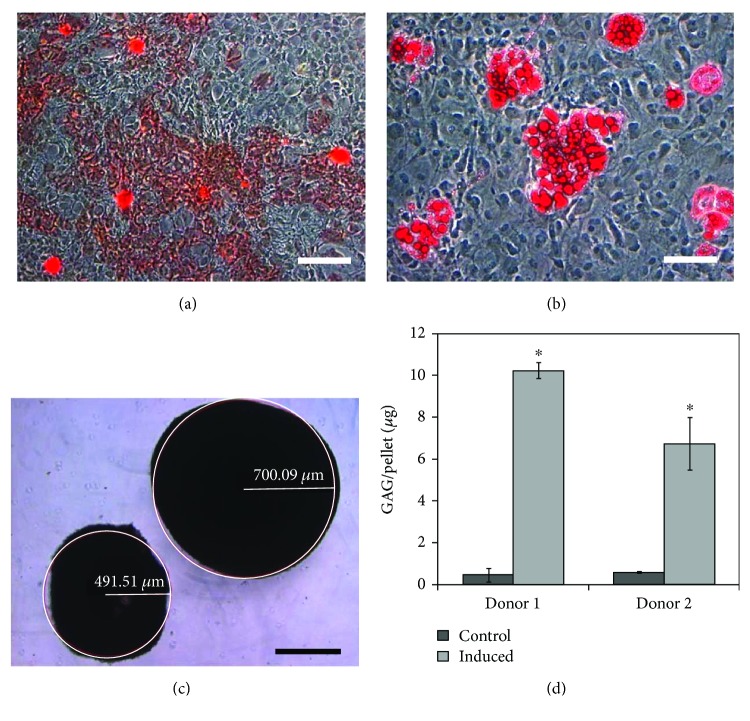
Multipotent differentiation potential of SF-MSCs cultured for 18 days in spinner flasks using the bead-to-bead transfer method. Multipotency was evaluated for cells isolated from both donor 1 and donor 2. Shown here are photomicrographs for donor 1, which are representative of the qualitative results obtained for donor 2. (a) Osteogenic differentiation of donor 1 SF-MSCs. Calcium deposition is an indication of osteogenesis and was detected using Alizarin Red. Photomicrograph was taken at 10x magnification. Scale bar represents 200 *μ*m. (b) Adipogenic differentiation of donor 1 SF-MSCs. Intracellular lipid droplet formation is an indication of adipogenesis and was detected using Oil Red O. Photomicrograph was taken at 20x magnification. Scale bar represents 100 *μ*m. (c) Chondrogenic differentiation of donor 1. Glycosaminoglycan (GAG) production is an indication of chondrogenesis and results in an increase in pellet size (right pellet was induced; left pellet was not induced (control)). Photomicrograph was taken at 5x magnification. Scale bar represents 500 *μ*m. (d) Quantitative chondrogenic differentiation analysis of donor 1 and donor 2 using the pellet culture method. 2.5 × 10^5^ cells/pellet were cultured in either 10% FBS DMEM (control) or chondrogenic induction medium (induced) for 28 days at 37°C and 5% CO_2_. GAG production was quantified using 1,9-dimethylmethlylene blue after digesting the pellets in papain solution. Data were collected in triplicate; error bars represent the standard deviation of the data. ^∗^*p* < 0.05 compared to the control.

Osteogenesis and adipogenesis were qualitatively verified by staining-induced cultures with Alizarin Red and Oil Red O, respectively, as shown in Figures 8(a) and 8(b). Two different analyses were performed to verify chondrogenesis. The first was to perform a size analysis on cell pellets which had been exposed to chondrogenic factors normally used to differentiate MSCs towards a chondrogenic lineage. Once induced, cells tend not to proliferate, so an increase in induced pellet size compared to an uninduced pellet of cells would indicate an upregulation of extracellular matrix production. For the cells grown on microcarriers in suspension culture, it was found that the induced pellets were significantly larger than the uninduced controls with an estimated radius of 700 *μ*m compared to 490 *μ*m, respectively (*p* < 0.05; see Figure 8(c)). This result showed that the cells maintained an ability to undergo chondrogenic differentiation even after being placed in suspension culture. To determine if the chondrogenic capacity of the cells grown in suspension culture was altered relative to cells cultured in traditional static tissue culture flasks, MSCs expanded for 18 days in static culture alongside the suspension cultures were also pelleted and then either chondrogenically induced or left uninduced. For these static culture cells, it was found that the induced pellets were significantly larger than the uninduced control values with an estimated radius of 690 *μ*m compared to 400 *μ*m, which is similar to the results found for cells from suspension culture. These results highlight that the chondrogenic capacity of the cells was not negatively impacted by being placed on microcarriers in suspension culture.

The second analysis related to chondrogenesis was to perform a GAG analysis on the pellets. For the cells grown in suspension culture, the average GAG content in the uninduced SF-MSC controls from donors 1 and 2 were 0.47 ± 0.31 *μ*g and 0.51 ± 0.05 *μ*g, respectively, whereas in the induced pellets, the GAG levels were significantly higher for both donor 1 and 2 at 10.24 ± 0.39 *μ*g and 6.74 ± 1.26 *μ*g, respectively (see Figure 8(d)). For the cells grown in static culture, the GAG levels in the induced controls were 9.94 ± 0.04 *μ*g and 8.89 ± 2.63 *μ*g for donors 1 and 2, respectively. The observed difference in differentiation potential between the two donors may be attributed to inherent donor-to-donor variability, or possibly due to the difference in donor age. Donor-to-donor variability in MSC populations is commonly reported in the literature [25, 54, 60]. In addition, it is generally believed that MSC function declines with age [61, 62], thereby contributing to any observed differences in behaviour between cells from different age donors, although the chondrogenic potential of synovium-derived MSCs has been shown to be age independent (De Bari et al., 2001).

## 4. Conclusions

The mesenchymal stem cells isolated from the synovial fluid of articulating joints have the potential to be used in cartilage repair strategies. However, the development of clinical applications will require large number of cells. This work clearly illustrated that microcarrier technology in scalable suspension culture bioreactors can provide the controlled environment required to expand synovial fluid-derived mesenchymal stem cells without compromising their defining characteristics, thereby providing a viable alternative to using tissue culture flasks. Moreover, this approach was found to be effective for cells from more than one donor. The ability to reproducibly support the expansion of stem cell populations from different donors is an important consideration when developing bioprocesses for clinical purposes.
